# Human Insulin-Like Growth Factor II mRNA-Binding Protein 3 (IMP3) and p16 Expression in Squamous Cell Carcinoma of the Head and Neck

**DOI:** 10.7759/cureus.104895

**Published:** 2026-03-09

**Authors:** Umasankary Calaisselvane, Kevin Manuel, Marie Moses Ambroise, Sithananda Kumar Venkatesan

**Affiliations:** 1 Pathology, Mahatma Gandhi Medical College and Research Institute, Sri Balaji Vidyapeeth (Deemed to be University), Puducherry, IND; 2 Pathology, Pondicherry Institute of Medical Sciences, Puducherry, IND; 3 Ear, Nose, and Throat (ENT), Pondicherry Institute of Medical Sciences, Puducherry, IND

**Keywords:** head and neck neoplasms, immunohistochemistry, imp3, p16, squamous cell carcinoma (scc)

## Abstract

Background

Head and neck squamous cell carcinoma (HNSCC) represents a substantial global health problem due to its considerable incidence and frequent presentation at advanced stages. The validation of biomarkers is essential to facilitate early detection and prognosis and inform targeted therapeutic interventions which can contribute to improved clinical outcomes. This study investigates the immunohistochemical expression patterns of two promising biomarkers: the oncofetal protein human insulin-like growth factor II mRNA-binding protein 3 (IMP3) and the tumour suppressor p16. IMP3, normally expressed during embryogenesis, is aberrantly re-expressed in malignancies and linked to tumour progression and poor outcomes. Conversely, p16 overexpression is frequently linked to human papillomavirus (HPV)-related carcinogenesis and is considered a favorable prognostic marker in HNSCC.

Materials and methods

A cross-sectional study of 50 HNSCC cases over 18 months evaluated IMP3 and p16 expression and correlated findings with clinicopathological variables (demographics, site, grade, stage, tobacco/alcohol use). Immunohistochemistry followed standard protocols with appropriate positive and negative controls. Statistical analyses used the chi-squared and Fisher's exact tests where applicable.

Results and conclusions

IMP3 was positive in 27 (54%) and p16 in six (12%) of HNSCC cases. The markers were mutually exclusive and showed significant associations with gender and tobacco use. IMP3 expression was higher in males (p < 0.05), while p16 correlated with specific risk factors, consistent with HPV-related pathways. Their mutual exclusivity suggests distinct molecular mechanisms in HNSCC. These differential patterns support IMP3 and p16 as complementary biomarkers for patient stratification and tailored management strategies. Further studies integrating molecular HPV status and long-term follow-up are needed to validate prognostic and therapeutic relevance.

## Introduction

Head and neck squamous cell carcinoma (HNSCC) constitutes over 90% of malignant tumours involving the oropharynx and represents the sixth most common cancer worldwide [1]. The global incidence of HNSCC has been steadily increasing, with approximately 890,000 new cases and 450,000 deaths reported annually [2]. In India, oral cancer ranks among the top three cancers, with age-adjusted incidence rates of 12.8 per 100,000 males and 5.8 per 100,000 females [3].

HNSCC arising from the oral cavity is a multifactorial disease with complex etiopathogenesis [4]. The principal risk factors include tobacco use, alcohol consumption, betel quid chewing, and infection with human papillomavirus (HPV) [5]. The molecular pathogenesis involves a series of genetic and epigenetic alterations that affects oncogenes, tumour suppressor genes, and DNA repair mechanisms [6].

Early diagnosis of oral cancer provides significant benefits by reducing mortality rates and treatment-related morbidity. However, the majority of HNSCC cases are diagnosed at advanced stages, resulting in poor prognosis with five-year survival rates of approximately 50-60% [7]. The development of reliable biomarkers for early detection, prognosis prediction, and treatment response monitoring remains a critical clinical need [8].

The oncofetal protein human insulin-like growth factor II mRNA-binding protein 3 (IMP3) plays essential roles in RNA metabolism, cell migration, and tumour progression. IMP3 is normally expressed during embryogenesis but is absent in most adult tissues, making it an attractive oncological biomarker [9]. Aberrant IMP3 expression has been reported in various malignancies, including lung, breast, gastric, and colorectal cancers [10].

The p16 protein, encoded by the CDKN2A gene, functions as a tumour suppressor by regulating cell cycle progression through the retinoblastoma pathway. p16 overexpression in HNSCC is often associated with HPV infection and correlates with improved prognosis [11]. However, the relationship between p16 expression and HPV status varies across different anatomical sites and populations [12].

Limited studies have investigated the combined expression patterns of IMP3 and p16 in HNSCC, particularly in the Indian population. Understanding their expression profiles and clinical correlations may provide insights into HNSCC pathogenesis and identify potential therapeutic targets [13,14].

## Materials and methods

Study design and patient selection

This cross-sectional study was conducted in the Department of Pathology, Pondicherry Institute of Medical Sciences, Puducherry, India, on 50 histopathologically confirmed cases of HNSCC. The study protocol was approved by the institute's Institutional Ethics Committee (approval number: RC/2020/110). The study period was carried out over a period of 18 months from January 2021 to June 2022. The study included both retrospective (January 2015 to December 2020) and prospective (January 2021 to June 2022) study periods. Cases were selected based on the following inclusion criteria: histopathologically confirmed diagnosis of HNSCC diagnosed between January 2015 and June 2022. Exclusion criteria included the following: (1) inadequate tissue in paraffin blocks for conducting immunohistochemistry (IHC) and (2) tissue with fixation artefacts. Patient demographics, tumour characteristics, staging information, and risk factor exposure history were extracted from medical records and pathology reports.

Tissue processing, histopathological evaluation, and immunohistochemical staining protocol

All tissue specimens were routinely processed and embedded in paraffin following standard protocols. Serial sections of 3-μm thickness were cut from representative tumour blocks and mounted on positively charged glass slides. Hematoxylin and eosin (H&E)-stained sections were reviewed by two independent pathologists to confirm the diagnosis and assess tumour characteristics including histological grade, depth of invasion, and presence of lymphovascular invasion (LVI). Tumour grading was performed according to the World Health Organization (WHO) classification system, categorizing tumours as well-differentiated (Grade I), moderately differentiated (Grade II), or poorly differentiated (Grade III) based on the degree of keratinization, nuclear pleomorphism, and mitotic activity. Tumour staging was determined using the American Joint Committee on Cancer (AJCC) 8th Edition TNM staging system. Manual IHC was performed on 3-µm formalin-fixed, paraffin-embedded sections using the EnVision FLEX High pH system (Agilent Technologies, Santa Clara, CA, USA). Sections on FLEX IHC slides were heated for 30 minutes at 60°C and then underwent heat-induced epitope retrieval using EnVision™ FLEX Target retrieval solution. The slides were washed with EnVision™ FLEX wash buffer, and endogenous peroxidase activity was blocked using EnVision™ FLEX Peroxidase-Blocking reagent. Ready-to-use mouse monoclonal primary antibodies against IMP3 (Clone: EP286; Master Diagnóstica S.L., Granada, Spain) and p16 (Clone: MX007; Master Diagnóstica S.L., Granada, Spain) were applied for one hour, followed by horseradish peroxidase (HRP)-linked secondary antibodies for 30 minutes. The detection method used was EnVision FLEX DAB chromogen, with hematoxylin counterstaining, water wash, and final mounting in dibutylphthalate polystyrene xylene (DPX).

Scoring and interpretation

**Figure 1:**
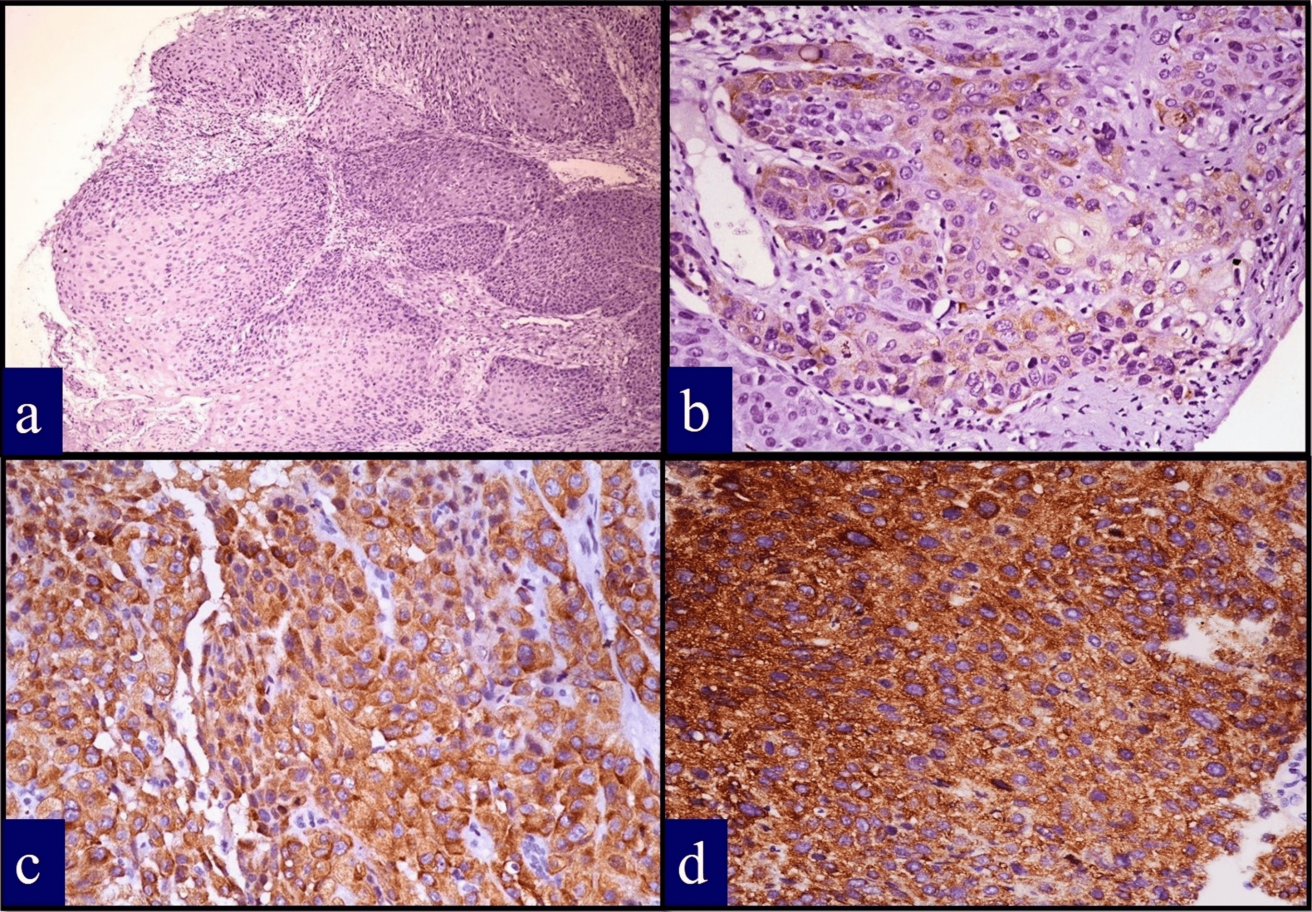
IMP3 expression (a) Negative expression with no staining in the cytoplasm of the tumour cells (×100). (b) Weak staining intensity in the cytoplasm of the tumour cells (×400). (c) Moderate staining intensity in the cytoplasm of the tumour cells (×400). (d) Marked staining intensity in the cytoplasm of the tumour cells (×400). IMP3: insulin-like growth factor II mRNA-binding protein 3

IMP3 immunostaining was evaluated based on cytoplasmic staining intensity and distribution. Scoring was performed using a semi-quantitative approach as shown in Figure 1: negative (0) (no staining or weak staining in <10% of tumour cells), positive (1+) (weak to moderate staining in 10-50% of tumour cells), and strongly positive (2+) (moderate to strong staining in >50% of tumour cells). For statistical analysis, cases were dichotomized into negative (score 0) and positive (scores 1+ and 2+) groups.

**Figure 2:**
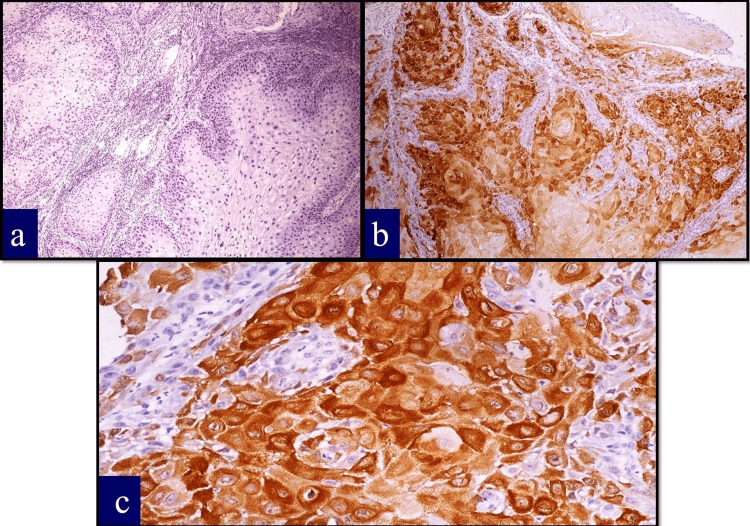
p16 expression (a) Negative expression with no staining in the nucleus and cytoplasm of the tumour cells (×100). (b) Strong staining intensity in the nucleus and cytoplasm of the tumour cells (×100). (c) Strong staining intensity in the nucleus and cytoplasm of the tumour cells (×400).

p16 immunostaining was assessed according to the established criteria for HPV surrogate marker interpretation as shown in Figure 2. Strong and diffuse nuclear and cytoplasmic staining in >70% of tumour cells was considered positive, while patchy, weak, or absent staining was classified as negative. Two independent pathologists performed the scoring, and discordant cases were resolved through consensus review.

Statistical analysis

Statistical analysis was performed using IBM SPSS Statistics for Windows, V. 20.0 (IBM Corp., Armonk, NY, USA). Descriptive statistics were calculated for all variables. Categorical variables were expressed as frequencies and percentages, while continuous variables were presented as mean ± standard deviation or median with interquartile range as appropriate. The association between IMP3 and p16 expression and clinicopathological parameters was evaluated using the chi-squared test or Fisher's exact test for categorical variables. Correlation between IMP3 and p16 expression was assessed using Spearman's rank correlation coefficient. Survival analysis was performed using the Kaplan-Meier method with the log-rank test for comparison between groups. Multivariate analysis was conducted using the Cox proportional hazards regression model to identify independent prognostic factors. A p-value of <0.05 was considered statistically significant for all analyses.

## Results

Patient demographics and tumour characteristics

A total of 50 histopathologically confirmed HNSCC cases were included. The mean patient age was 57.8 ± 12.2 years (35-91), with a male predominance (37, 74%). The oral cavity was the most common primary site (27, 54%). Risk factors were identified in 34 (68%) patients, with predominantly chewing habits (23, 46%). Sexual history was assessed in 11 prospective cases, all denying multiple partners; records for retrospective cases lacked such data.

Moderately differentiated squamous cell carcinoma (SCC) was the most frequent histologic subtype (28, 56%), followed by well-differentiated (11, 22%) and poorly differentiated (8, 16%) tumours. Three cases (two papillary, one sarcomatoid) were ungraded. LVI and perineural invasion (PNI) were each present in five cases. Adjacent mucosa showed epithelial hyperplasia and benign epithelium (38 cases), mild to moderate dysplasia (11 cases), and severe dysplasia/carcinoma in situ (six cases). TNM data were incomplete; the T stage was available for 39 cases and N stage for 33 cases.

IMP3 and p16 expression analysis

Among 50 HNSCC cases, 27 (54%) were IMP3 positive and six (12%) were p16 positive. Table 1 shows the immunohistochemical expression of IMP3 and p16 in HNSCC included in the study. IMP3 expression showed a significant association with male gender (24, 88.9%; p = 0.009), whereas p16 expression correlated significantly with female gender (4, 66.7%; p = 0.033) and tobacco chewing (5, 83.3%; p = 0.010).

**Table 1:** Immunohistochemical expression of IMP3 and p16 in HNSCC IMP3: insulin-like growth factor II mRNA-binding protein 3; HNSCC: head and neck squamous cell carcinoma

	p16 negative (n (%))	p16 positive (n (%))	Total (n (%))
IMP3 negative	18 (36%)	5 (10%)	23 (46%)
IMP3 positive	26 (52%)	1 (2%)	27 (54%)
Total	44 (88%)	6 (12%)	50 (100%)

Table 2 highlights the association of IMP3 and p16 with gender and risk habits. IMP3 showed no significant association with patient habits or tumour site. The oral cavity was the most common site among both IMP3-positive (14, 51.9%) and p16-positive (5, 18.5%) tumours. Neither IMP3 nor p16 expression correlated significantly with histological grade (p = 0.083 and p = 0.820, respectively), though IMP3 tended to be negative in well-differentiated and positive in poorly differentiated SCC.

**Table 2:** Association of IMP3 and p16 expression with gender and risk habits IMP3: insulin-like growth factor II mRNA-binding protein 3

Characteristics		IMP3 positive (n (%))	IMP3 negative (n (%))	P-value	p16 positive (n (%))	p16 negative (n (%))	P-value
Gender	Male	24 (88.9%)	13 (56.5%)	0.009	2 (33.3%)	35 (79.5%)	0.033
	Female	3 (11.1%)	10 (43.5%)		4 (66.7%)	9 (20.5%)	
Alcohol abuse	Present	12 (44.4%)	6 (26.1%)	0.178	1 (16.7%)	17 (38.6%)	0.339
	Absent	15 (55.6%)	17 (73.9%)		5 (83.3%)	27 (61.4%)	
Cigarette smoking	Present	13 (48.1%)	7 (30.4%)	0.203	2 (33.3%)	18 (40.9%)	1.000
	Absent	14 (51.9%)	16 (69.6%)		4 (66.7%)	26 (59.1%)	
Tobacco chewing	Present	9 (33.3%)	7 (30.4%)	0.827	5 (83.3%)	11 (25%)	0.010
	Absent	18 (66.7%)	16 (69.6%)		1 (16.7%)	33 (75%)	
Paan chewing	Present	2 (7.4%)	2 (8.7%)	1.000	1 (16.7%)	3 (6.8%)	0.411
	Absent	25 (92.6%)	21 (91.3%)		5 (83.3%)	41 (93.2%)	
Betel nut chewing	Present	6 (22.2%)	3 (13%)	0.479	3 (50%)	6 (13.6%)	0.063
	Absent	21 (77.8%)	20 (87%)		3 (50%)	38 (86.4%)	
Beedi smoking	Present	1 (3.7%)	3 (13%)	0.322	2 (33.3%)	2 (4.5%)	0.066
	Absent	26 (96.3%)	20 (87%)		4 (66.7%)	42 (95.5%)	

**Figure 3:**
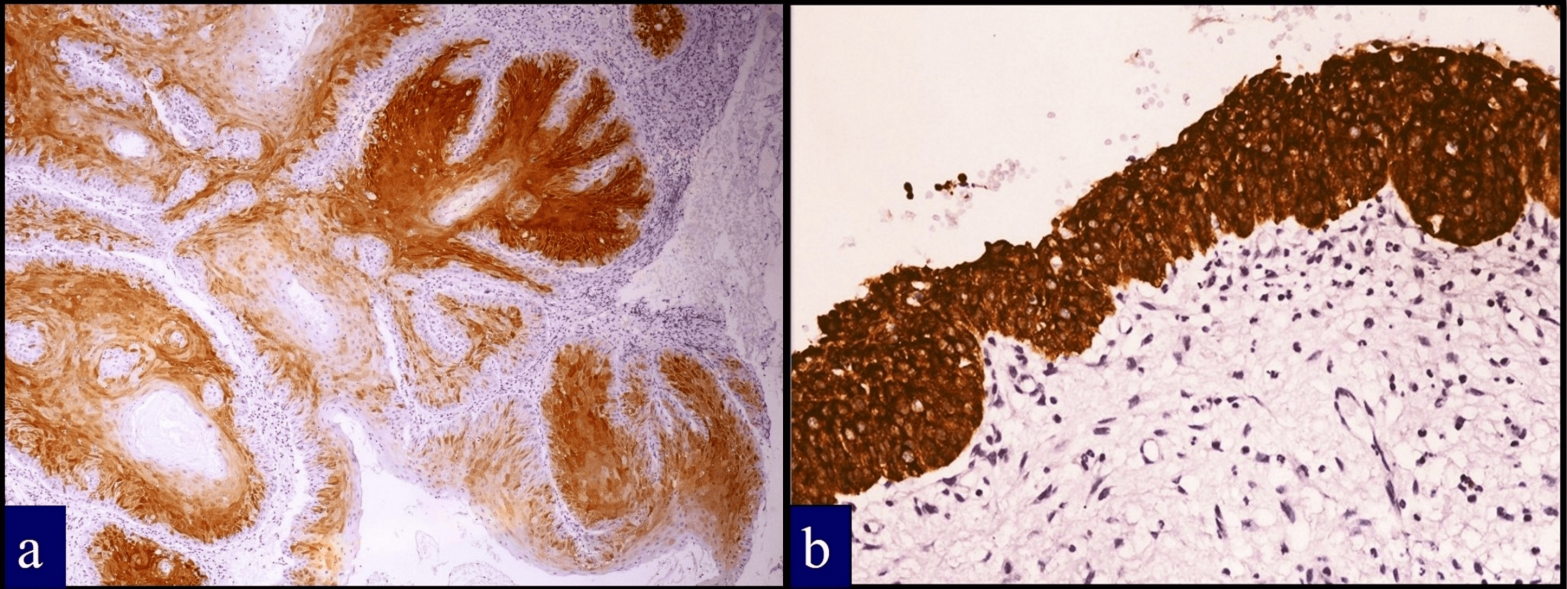
IMP3 expression in special cases (a) Strong IMP3 expression in a case of papillary squamous cell carcinoma (×100). (b) Strong IMP3 expression in a case of carcinoma in situ (×400). IMP3: insulin-like growth factor II mRNA-binding protein 3

One of two papillary SCCs was IMP3 positive (Figure 3a), while both were p16 negative. Adjacent benign epithelium and epithelial hyperplasia were IMP3 negative, with weak staining in one of 11 cases that showed mild/moderate dysplasia. Strong IMP3 expression was observed in five (83%) cases of severe dysplasia/carcinoma in situ (Figure 3b). p16 showed faint staining in two benign and five hyperplastic cases and strong diffuse expression in one of six severe dysplasia/carcinoma in situ cases. Neither IMP3 nor p16 expression showed significant correlation with T stage (p = 0.116 and p = 0.640) or N stage (p = 0.223 and p = 0.590).

Correlation between IMP3 and p16 expression

Out of 50 HNSCC cases, only one (2%) co-expressed p16 and IMP3. Both markers showed gender-related significance but no association with site, grade, invasion, or stage. The sarcomatoid SCC was negative for both markers.

## Discussion

HNSCC remains a devastating disease with high morbidity and mortality rates. Early evaluation and diagnosis of HNSCC reduces the incidence of invasive carcinomas and mortality rate and also facilitates the treatment of smaller lesions.

In the present study, males constituted the majority (37, 74%), with the oral cavity being the most common site (27, 54%). Similar findings have been reported in several Indian studies linking oral cavity cancers to risk factors such as tobacco chewing, paan chewing, betel nut use, smoking, and alcohol consumption [15-18]. However, Ralli et al. reported the oropharynx as the predominant site (60, 80%) [19]. In contrast, studies from Europe and Western countries consistently identify the oropharynx (particularly the base of the tongue and tonsil) as the most common site of HNSCC [20-24]. The rising incidence of oropharyngeal cancers in these regions has been attributed to HPV-16 infection and high-risk sexual behaviours, including oral sex and multiple sexual partners [25-27]. Indian studies seldom address sexual practices due to cultural sensitivities, and such behaviours are generally less prevalent compared to Western populations. This likely accounts for the lower incidence of HPV-associated oropharyngeal cancers in India, where oral cavity cancers remain the dominant subtype of HNSCC, reflecting distinct lifestyle and sociocultural risk profiles [28].

IMP3 expression is studied in normal and malignant tissues and is regarded as a useful prognostic marker and potential biomarker in various human solid tumours, like renal cell carcinoma, endometrial carcinoma, cervical adenocarcinoma, malignant melanoma, urothelial carcinoma, gastric adenocarcinoma, pancreatic adenocarcinoma, hepatocellular carcinoma, and triple-negative breast carcinoma [29,30]. Despite this broad spectrum of studies, the expression of IMP3 in HNSCCs has been addressed only in a limited number of investigations.

Table 3 summarizes IMP3 expression in various HNSCC studies. Both the present study and that of Riener et al. considered moderate to strong staining in ≥25% of tumour cells as positive [24]. Our results were closely matched with those of Riener et al. [24]. Lin et al. applied the same cut-off and reported 54.8% positivity [31]. A meta-analysis on IMP3 in solid tumours noted considerable heterogeneity in the cut-off definitions for high expression [29]. In our study, IMP3 showed variable staining intensities, suggesting that a combined scoring system based on both percentage positivity and intensity may yield greater accuracy; however, optimal cut-off values in HNSCC require further validation with quantitative polymerase chain reaction (qPCR).

**Table 3:** IMP3 expression in various studies Time period means the period when the biopsies included in each study were obtained. HNSCC: head and neck squamous cell carcinoma; OSCC: oral squamous cell carcinoma

S. no.	Author	Place of study and time period	Site	Sample size	IMP3 positivity	Criteria for positivity
1	Riener et al. [24]	Erlangen, Germany (1998-2015)	HNSCC	156	51.90%	Tumours were scored as 0, 1+, 2+, and 3+
						2+ and 3+ were considered positive
						2+ (moderate to strong staining in 25-50% of tumour cells)
						3+ (moderate to strong staining in >50% of tumour cells)
2	Chen et al. [29]	Guangdong, South China (2000-2005)	Laryngeal carcinoma	227	92%	IMP3 negative (no staining of tumour cells)
						IMP3 positivity: focal staining 1+ (<25% of tumour cells)
						IMP3 positivity: moderate staining 2+ (25-50% of tumour cells)
						IMP3 positivity: diffuse staining 3+ (>50% of tumour cells)
3	Lin et al. [31]	Taipei, Taiwan (1997-2004)	OSCC	148	54.8%	Tumours were scored as 1+, 2+, 3+, and 4+
						1+ (<25% of tumour cells)
						2+ (25-50% of tumour cells)
						3+ (50-75% of tumour cells)
						4+ (≥75% of tumour cells (high expression))
4	Present study	Puducherry, India (2015-2022)	HNSCC	50	54%	Similar criteria as Riener et al.

IMP3 expression in adjacent normal epithelium, hyperplasia, and dysplasia in our study was consistent with Chen et al. [32]. Its expression in premalignant lesions merits further study, particularly for diagnostic biopsies. Morimatsu et al. observed IMP3 positivity in 71.8% of pancreatic intraductal papillary mucinous neoplasms (IPMNs) with high-grade dysplasia and in 81.3% with invasive carcinoma, but not in low- or intermediate-grade dysplasias [33]. Collectively, these findings suggest IMP3 may help distinguish malignant from non-malignant lesions.

p16 is a tumour suppressor gene involved in cell cycle regulation and serves as a surrogate endpoint of HPV infection in oropharyngeal squamous cell carcinoma (OPSCC) [24]. Most Indian studies report p16 positivity below 20%, with wide variation attributed to differing scoring criteria, biopsy sites, and associated risk factors such as sexual habits. Pandey et al. observed diffuse positivity (30-85% of labelled cells with strong positivity) only in 19 (19%) cases [15]. Ralli et al. reported the highest p16 positivity among oropharyngeal cancers using a graded scoring system where p16 was graded as negative (0-5% of nuclei and cytoplasm positive), sporadic (5-10% of nuclei and cytoplasm with weak and scattered positivity), focal (>10-30% of labelled nuclei and cytoplasm strongly positive, spreading in one tissue area), and diffuse (>30-85% of labelled cells with strong positivity) [19]. The College of American Pathologists (CAP) recommends strong, diffuse nuclear and cytoplasmic staining in >70% of tumour cells as the positivity criterion [34]. The majority of the Western studies have used a similar cut-off. Riener et al. used a 50% threshold but noted 80-100% staining in all positive cases [24].

Table 4 compares p16 expression in both Western and Indian studies. The relative percentage of oropharyngeal carcinomas is higher in the HNSCC studies due to the increasing incidence of HPV-associated oropharyngeal cancers in the Western world. p16 expression is 53% in OPSCC and higher compared to the present study. There were very few oropharyngeal carcinomas in our study, and the relative percentage of OPSCC is lower in other Indian HNSCC studies. In the study by Biesaga et al., the overall p16 positivity is 73.5%. The p16 positivity was 84% in the oropharynx [21]. Interestingly, the p16 positivity in oropharyngeal carcinomas was considerably lower in Indian studies [35,36].

**Table 4:** p16 expression in both Western and Indian studies Time period means the period when the biopsies included in each study were obtained. OSCC: oral squamous cell carcinoma; HNSCC: head and neck squamous cell carcinoma; OPSCC: oropharyngeal squamous cell carcinoma; SCC: squamous cell carcinoma

S. no.	Author	Place of study and time period	Sample size	Site	p16 positivity	Criteria for positivity
1	Pandey et al. [15]	Bahraich, Uttar Pradesh, India (2017-2018)	100	HNSCC	60%	Nuclear and cytoplasmic positivity in >5% tumour cells
2	Naz et al. [18]	New Delhi, India (2022)	800	OSCC	17.37%	Cases with positive nuclear staining in >75% cells
3	Ralli et al. [19]	Rohtak, Haryana, India (2012-2014)	75	HNSCC	78.67%	Nuclear and cytoplasmic positivity in >5% tumour cells
4	Biesaga et al. [21]	Cracow, Poland (2007-2014)	109	HNSCC	73.5%	>75% of tumour cells with nuclear and cytoplasmic staining or >50% staining with >25% confluent positive staining areas
5	Arsa et al. [22]	Bangkok, Thailand (2007-2018)	662	HNSCC	10.9%	>70% of tumour cells with nuclear and cytoplasmic staining
6	Riener et al. [24]	Erlangen, Germany (1998-2015)	427	HNSCC	34.6%	>50% of positive staining cells with nuclear and cytoplasmic stain
7	Fakhry et al. [34]	San Francisco, USA (1995-2012)	860	HNSCC	16.74%	>70% strong and diffuse nuclear and cytoplasmic staining
8	Bhosale et al. [35]	Mumbai, Maharashtra, India (2016)	427	HNSCC	2.1%	Nuclear and cytoplasmic positivity in >70% of tumour cells
9	Raphael et al. [36]	Kottayam, Kerala, India (2019-2020)	210	OSCC and OPSCC	4%	Diffuse and strong nuclear and cytoplasmic staining in >70% of malignant cells
10	Agarwal et al. [37]	New Delhi, India (2017-2019)	50	OSCC	30%	Multiplicative score obtained from the proportion of malignant cell staining and intensity
11	Ramshankar et al. [38]	Chennai, Tamil Nadu, India (2007)	156	Tongue SCC	15.38%	>50% of tumour cells with intense nuclear and cytoplasmic staining
12	Present study	Puducherry, India (2015-2022)	50	HNSCC	12%	Diffuse and strong nuclear and cytoplasmic staining in >70% of malignant cells

IMP3 is an aggressive marker, whereas p16 is considered a surrogate marker for high-risk HPV. p16 expression is associated with a good prognosis. Hence, we aimed to evaluate the diagnostic and clinical utility of combining IMP3 and p16 expression in HNSCC. To our thorough search till date, there was only one study that evaluated IMP3 and p16 expression in HNSCC by Riener et al. [24]. There were no other similar studies with the combination of these two immunostains in HNSCC. Also, there are many Indian HNSCC studies on p16. However, there were no Indian studies on IMP3. Table 5 highlights the comparison of the association of IMP3 and p16 expression in a study done by Riener et al. [24] and the present study.

**Table 5:** Comparison of the association of IMP3 and p16 expression in a study done by Riener et al. and the present study IMP3: insulin-like growth factor II mRNA-binding protein 3

IMP3 and p16 expression	Riener et al. (n (%))	Present study (n (%))
IMP3+ p16+	30 (19.2%)	1 (2%)
IMP3+ p16-	51 (32.7%)	26 (52%)
IMP3- p16+	24 (15.4%)	5 (10%)
IMP3- p16-	51 (32.7%)	18 (36%)

In our study, 27 (54%) patients were IMP3 positive, and 23 (46%) of them were IMP3 negative. The majority of them with IMP3 expression showed strong diffuse positivity with a score of 3+. Only six (12%) patients were positive for p16, and 44 (88%) of them were negative for p16.

Riener et al. did not find the association between IMP3 and p16 expression to be statistically significant (p = 0.61) [24]. Our study showed IMP3 positivity in 54% (n = 27) of tumours and p16 positivity in 12% (n = 6) of HNSCC. About 52% (n = 26) of tumours are positive only for IMP3, and 10% (n = 5) are positive only for p16. Only 2% (n = 1) were positive for both. In our study, IMP3 and p16 expression seem to be mutually exclusive. A trend towards a negative association between IMP3 expression and HPV infection was observed. The degree of IMP3 expression appears similar in both studies. p16 was expressed in 61.7% of OPSCC cases compared to 41.3% of other HNSCC. The p16 expression is less compared to the study by Riener et al. [24], probably due to the lack of HPV-associated OPSCC in our cohort. Evaluation of HPV status would have yielded more information in our study. p16 overexpression can occur in the absence of detectable HPV E7 expression. This may be related to the alteration of the Rb gene or due to an innate increase in p16 expression. Inactivation of the Rb gene by mutations or deletion, p16 amplification, and mutations of histone H3 lysine 36 methyltransferase genes can cause p16 overexpression. The molecular basis for p16 overexpression in HPV-negative cases is still not clear and requires further studies [39].

In our study, we found a significant association between p16 expression and tobacco chewing (p = 0.01) but not with other risk habits like smoking and alcohol. Studies by Pandey et al., Ralli et al., and Agarwal et al. showed no significant association between p16 expression and risk habits like alcohol and smoking [15,19,37]. In India, tobacco chewing in various forms remains the commonest risk factor in the causation of oral cancers in youth as well as in adults. Smokeless tobacco can be equally hazardous as smoking cigarettes. Smokeless tobacco does not appear to be a risk factor in Western studies. The combined effect of alcohol, tobacco, and risk habits like tobacco, paan, and betel nut chewing needs to be investigated. p16-positive OPSCC of smokers showed clinical, imaging, and prognostic differences in comparison with p16-positive OPSCC of non-smokers. The p16-positive tumours in smokers were more aggressive [40]. Western studies have found p16 overexpression to be associated with low tobacco and alcohol consumption in OPSCC, whereas the results are variable for non-oropharyngeal carcinomas. Our study suggests that p16 expression can also be due to the effect of smokeless tobacco.

Indian studies by Pandey et al. and Ralli et al. demonstrated a significant p16 association with abnormal sexual habits. However, HPV testing was not carried out in them. We did not evaluate the association with abnormal sexual habits [15,19].

The association of p16 expression with HPV infection has been analysed in a few Indian studies. Sannigrahi et al. (Northern India) showed HPV DNA positivity in only 29.7% of HNSCC cases, and only 6.19% of them were positive for both HPV and p16. p16-positive cases had a greater viral load than those that were negative [41]. Ramshankar et al. studied 156 early-stage tongue cancers where HPV DNA prevalence was found in 81 cases and HPV 16 DNA in 69 cases. He concluded that p16 can be used in tongue cancers to predict poor prognosis and that HPV probably does not appear to play a significant role in oral tongue cancers since transcriptional activity in the form of p16 expression was absent in the majority of cases [38].

An Asian study by Arsa et al. showed a significant association between HPV and p16 expression in OPSCC but not with non-oropharyngeal carcinomas. The sensitivities of IHC for detecting p16 in patients with OPSCC and non-oropharyngeal carcinomas were 80% and 25%, and discordance rates of HPV/p16 status were 23% and 7%, respectively [22]. Further studies are required to determine the validity of p16 as a surrogate marker for HPV in non-oropharyngeal carcinomas. This is especially important in Asian countries. Moreover, p16 status as a surrogate marker for HPV-associated OPSCC might be more effective in Western countries with a high prevalence of p16-positive/HPV-associated OPSCC. It may not be so effective in Asian countries.

This study had several limitations. The majority of the cases included were wedge biopsies, incision biopsies, and endoscopic punch biopsies. T and N stage evaluation was not available for all the cases. HPV detection was not undertaken, limiting the assessment of its causal contribution. Future studies can resolve these limitations.

## Conclusions

This study demonstrates the clinicopathological profile of HNSCC in our cohort, with a clear male predominance. The oral cavity has been the most frequently affected site with tobacco-related habits emerging as the principal risk factors. IMP3 expression was identified in over half of the cases and was notably absent in benign and mildly to moderately dysplastic squamous epithelium while being retained in severe dysplasia and carcinoma in situ. These findings support the utility of IMP3 as an adjunctive marker in distinguishing malignant from non-malignant squamous lesions of the head and neck, although it does not reliably separate carcinoma in situ from invasive carcinoma.

p16 expression was observed in a smaller subset of tumours and demonstrated a mutually exclusive pattern with IMP3 in most cases. While both markers showed significant associations with gender and p16 additionally with tobacco chewing, neither correlated with histological grade or tumour stage. IMP3 appears to have diagnostic value in the evaluation of HNSCC, whereas the limited expression of p16 in this series underscores the need for further studies to clarify its role within different etiologic and patient populations.

## References

[1] Leemans CR, Snijders PJ, Brakenhoff RH (2018). The molecular landscape of head and neck cancer. Nat Rev Cancer.

[2] Siegel RL, Miller KD, Fuchs HE, Jemal A (2022). Cancer statistics, 2022. CA Cancer J Clin.

[3] Warnakulasuriya S (2009). Global epidemiology of oral and oropharyngeal cancer. Oral Oncol.

[4] Blot WJ, McLaughlin JK, Winn DM (1988). Smoking and drinking in relation to oral and pharyngeal cancer. Cancer Res.

[5] Hashibe M, Brennan P, Chuang SC (2009). Interaction between tobacco and alcohol use and the risk of head and neck cancer: pooled analysis in the International Head and Neck Cancer Epidemiology Consortium. Cancer Epidemiol Biomarkers Prev.

[6] (2015). Comprehensive genomic characterization of head and neck squamous cell carcinomas. Nature.

[7] Marur S, Forastiere AA (2016). Head and neck squamous cell carcinoma: update on epidemiology, diagnosis, and treatment. Mayo Clin Proc.

[8] Vigneswaran N, Williams MD (2014). Epidemiologic trends in head and neck cancer and aids in diagnosis. Oral Maxillofac Surg Clin North Am.

[9] Bell JL, Wächter K, Mühleck B, Pazaitis N, Köhn M, Lederer M, Hüttelmaier S (2013). Insulin-like growth factor 2 mRNA-binding proteins (IGF2BPs): post-transcriptional drivers of cancer progression?. Cell Mol Life Sci.

[10] Köbel M, Xu H, Bourne PA (2009). IGF2BP3 (IMP3) expression is a marker of unfavorable prognosis in ovarian carcinoma of clear cell subtype. Mod Pathol.

[11] Ang KK, Harris J, Wheeler R (2010). Human papillomavirus and survival of patients with oropharyngeal cancer. N Engl J Med.

[12] Rischin D, Young RJ, Fisher R (2010). Prognostic significance of p16INK4A and human papillomavirus in patients with oropharyngeal cancer treated on TROG 02.02 phase III trial. J Clin Oncol.

[13] Westra WH (2009). The changing face of head and neck cancer in the 21st century: the impact of HPV on the epidemiology and pathology of oral cancer. Head Neck Pathol.

[14] Gillison ML, Chaturvedi AK, Anderson WF, Fakhry C (2015). Epidemiology of human papillomavirus-positive head and neck squamous cell carcinoma. J Clin Oncol.

[15] Pandey P, Ralli M, Dixit A, Agarwal S, Chaturvedi V, Sawhney A, Agarwal R (2021). Assessment of immunohistochemical expression of p16 in head and neck squamous cell carcinoma and their correlation with clinicopathological parameters. J Oral Maxillofac Pathol.

[16] Chauhan R, Trivedi V, Rani R, Singh U (2022). A study of head and neck cancer patients with reference to tobacco use, gender, and subsite distribution. South Asian J Cancer.

[17] Gheit T, Anantharaman D, Holzinger D (2017). Role of mucosal high-risk human papillomavirus types in head and neck cancers in central India. Int J Cancer.

[18] Naz F, Verma H, Tanveer N, Sudheer AK, Kakkar A, Tanwar P (2022). Demographic profile of p16 immunopositive and HPV DNA PCR positive oral squamous cell carcinoma in a large cohort of Indian patients. Asian Pac J Cancer Prev.

[19] Ralli M, Singh S, Yadav SP, Sharma N, Verma R, Sen R (2016). Assessment and clinicopathological correlation of p16 expression in head and neck squamous cell carcinoma. J Cancer Res Ther.

[20] van Monsjou HS, van Velthuysen ML, van den Brekel MW, Jordanova ES, Melief CJ, Balm AJ (2012). Human papillomavirus status in young patients with head and neck squamous cell carcinoma. Int J Cancer.

[21] Biesaga B, Mucha-Małecka A, Janecka-Widła A (2018). Differences in the prognosis of HPV16-positive patients with squamous cell carcinoma of head and neck according to viral load and expression of P16. J Cancer Res Clin Oncol.

[22] Arsa L, Siripoon T, Trachu N (2021). Discrepancy in p16 expression in patients with HPV-associated head and neck squamous cell carcinoma in Thailand: clinical characteristics and survival outcomes. BMC Cancer.

[23] Pakdel F, Farhadi A, Pakdel T, Andishe-Tadbir A, Alavi P, Behzad-Behbahani A, Ashraf MJ (2021). The frequency of high-risk human papillomavirus types, HPV16 lineages, and their relationship with p16INK4a and NF-κB expression in head and neck squamous cell carcinomas in Southwestern Iran. Braz J Microbiol.

[24] Riener MO, Hoegel J, Iro H, Hartmann A, Agaimy A (2017). IMP3 and p16 expression in squamous cell carcinoma of the head and neck: a comparative immunohistochemical analysis. Oncol Lett.

[25] Scott-Wittenborn N, D'Souza G, Tewari S (2022). Prevalence of human papillomavirus in head and neck cancers at tertiary care centers in the United States over time. Cancer.

[26] Castellsagué X, Alemany L, Quer M (2016). HPV involvement in head and neck cancers: comprehensive assessment of biomarkers in 3680 patients. J Natl Cancer Inst.

[27] Ford K, Sohn W, Lepkowski J (2002). American adolescents: sexual mixing patterns, bridge partners, and concurrency. Sex Transm Dis.

[28] Joshi B, Chauhan S (2011). Determinants of youth sexual behaviour: program implications for India. East J Med.

[29] Chen L, Xie Y, Li X (2017). Prognostic value of high IMP3 expression in solid tumors: a meta-analysis. Onco Targets Ther.

[30] Maržić D, Marijić B, Braut T (2021). IMP3 protein overexpression is linked to unfavorable outcome in laryngeal squamous cell carcinoma. Cancers (Basel).

[31] Lin CY, Chen ST, Jeng YM (2011). Insulin-like growth factor II mRNA-binding protein 3 expression promotes tumor formation and invasion and predicts poor prognosis in oral squamous cell carcinoma. J Oral Pathol Med.

[32] Chen K, Cornejo KM, Ye W, Wu Q, Liang J, Jiang Z (2013). Oncofetal protein IMP3: a new diagnostic biomarker for laryngeal carcinoma. Hum Pathol.

[33] Morimatsu K, Aishima S, Yamamoto H (2013). Insulin-like growth factor II messenger RNA-binding protein-3 is a valuable diagnostic and prognostic marker of intraductal papillary mucinous neoplasm. Hum Pathol.

[34] Fakhry C, Lacchetti C, Rooper LM (2018). Human papillomavirus testing in head and neck carcinomas: ASCO clinical practice guideline endorsement of the College of American Pathologists Guideline. J Clin Oncol.

[35] Bhosale PG, Pandey M, Desai RS, Patil A, Kane S, Prabhash K, Mahimkar MB (2016). Low prevalence of transcriptionally active human papilloma virus in Indian patients with HNSCC and leukoplakia. Oral Surg Oral Med Oral Pathol Oral Radiol.

[36] Raphael R, Priya PV, Anju CK, Sankar S (2021). p16 expression and clinicopathological features of oral and oropharyngeal squamous cell carcinoma. Int J Res Med Sci.

[37] Agarwal VK, Sharma R, Gahlot G, Arnav A (2021). Clinical and histopathological correlation of p16 and p53 expression in oral cancer. Indian J Surg Oncol.

[38] Ramshankar V, Soundara VT, Shyamsundar V, Ramani P, Krishnamurthy A (2014). Risk stratification of early stage oral tongue cancers based on HPV status and p16 immunoexpression. Asian Pac J Cancer Prev.

[39] Wang H, Zhang Y, Bai W (2020). Feasibility of immunohistochemical p16 staining in the diagnosis of human papillomavirus infection in patients with squamous cell carcinoma of the head and neck: a systematic review and meta-analysis. Front Oncol.

[40] Trinh JM, Thomas J, Salleron J, Henrot P (2021). Differences in clinical and imaging characteristics between p16-positive non-smokers and p16-positive smokers or p16-negative patients in oropharyngeal carcinoma. Sci Rep.

[41] Sannigrahi MK, Singh V, Sharma R, Panda NK, Radotra BD, Khullar M (2016). Detection of active human papilloma virus-16 in head and neck cancers of Asian North Indian patients. Oral Dis.

